# Supplementary material for: Berberine Photo-Activation Potentiates Cytotoxicity in Human Astrocytoma Cells through Apoptosis Induction
Source: J Pers Med. 2021 Sep 22;11(10):942. doi: 10.3390/jpm11100942 (PMC8541605; doi:10.3390/jpm11100942)
Supplement: Supplementary file 1 [file jpm-11-00942-s001.zip › jpm-1351510-supplementary.pdf]

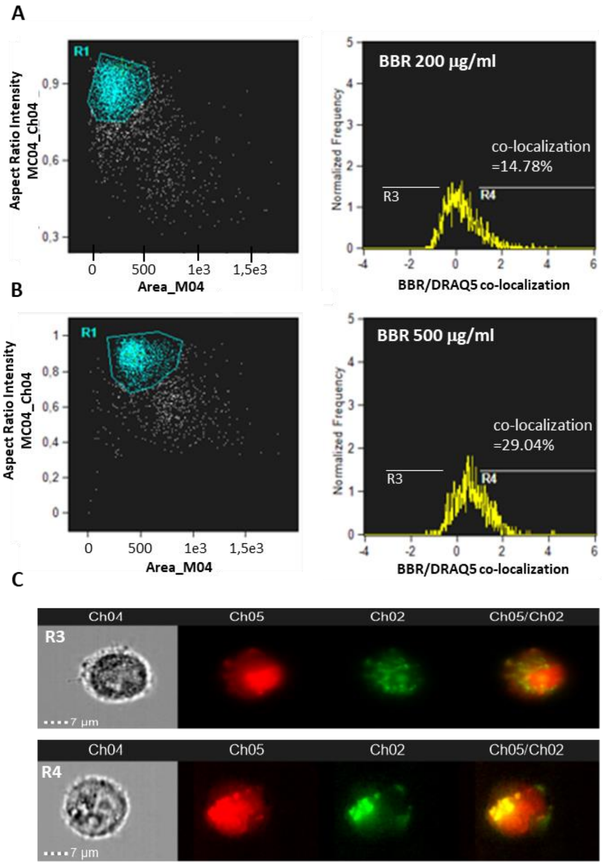

Figure S1. BBR nuclear localization. T98G cells were incubated with different BBR concentrations (i.e. 200 or 500 µg/ml for 4 hours and analysed after 24 hours p.t., panels **A** and **B**). Before trypsinization, cells were incubated at room temperature for 15 minutes with DRAQ5 nuclear staining dye (Invitrogen) and analysed by flow cytometry (Amnis ImageStream) at 60X magnification, excited at 488 (Ch02) and 642 nm (Ch05) for BBR and DRAQ5, respectively. Data was analysed using Ideas «Nuclear co-localization» tool reporting the percentages of R4 gated cells with BBR-DRAQ5 co-localized signals. Representative images of R3 and R4 gated cells are reported (**C**). R2 gates (not shown) were used to select focused cells.

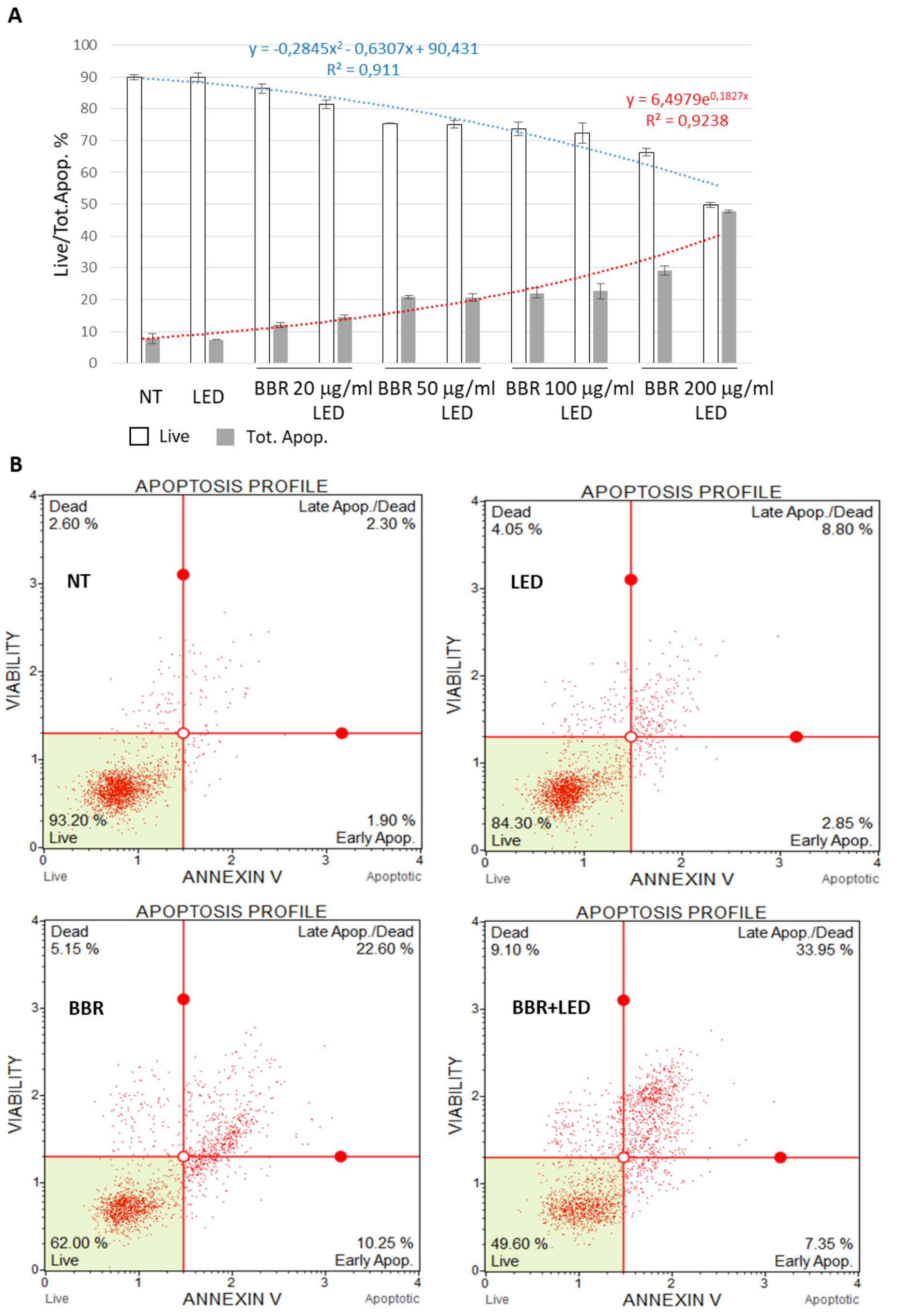

Figure S2. Live and apoptotic cells trends in BBR-PDT assays. T98G cells were assayed at different BBR concentrations (i.e. 20-50-100-200 μg/ml for 4 hours) and/or following led stimulation (4 minutes). After 24 hours p.t., cytofluorimetric evaluation of apoptosis by Annexin V and Cell Dead kit (Muse, Luminex) was performed evaluating 2000 cells for each treatment. Histogram reported the averages of live *vs* total apoptotic cells (i.e. early and late apoptotic) of two independent experiments. Trends and respective equations are reported (**A**). Representative Annexin V cytometric plots of 200 μg/ml BBR treatment with/without led stimulation (**B**).

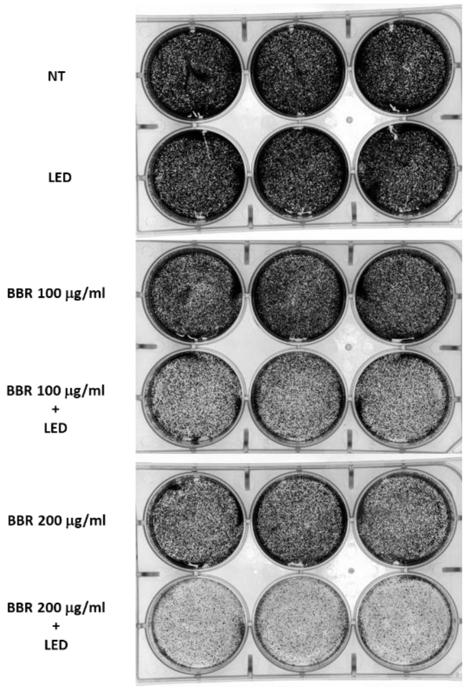

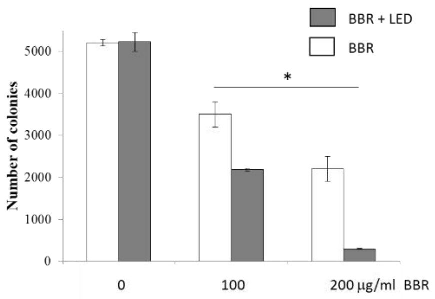

Figure S3. BBR-PDT clonogenic assay. T98G (10^4 cells) were trypsinized after the indicated treatments and grown into 6-well plates for two weeks. Colonies were stained with crystal violet and their numbers reported in the histogram below. Each experiment was performed in triplicate. Asterisk indicated statistical significance compared to untreated cells ($p<0.05$, Anova One-way).

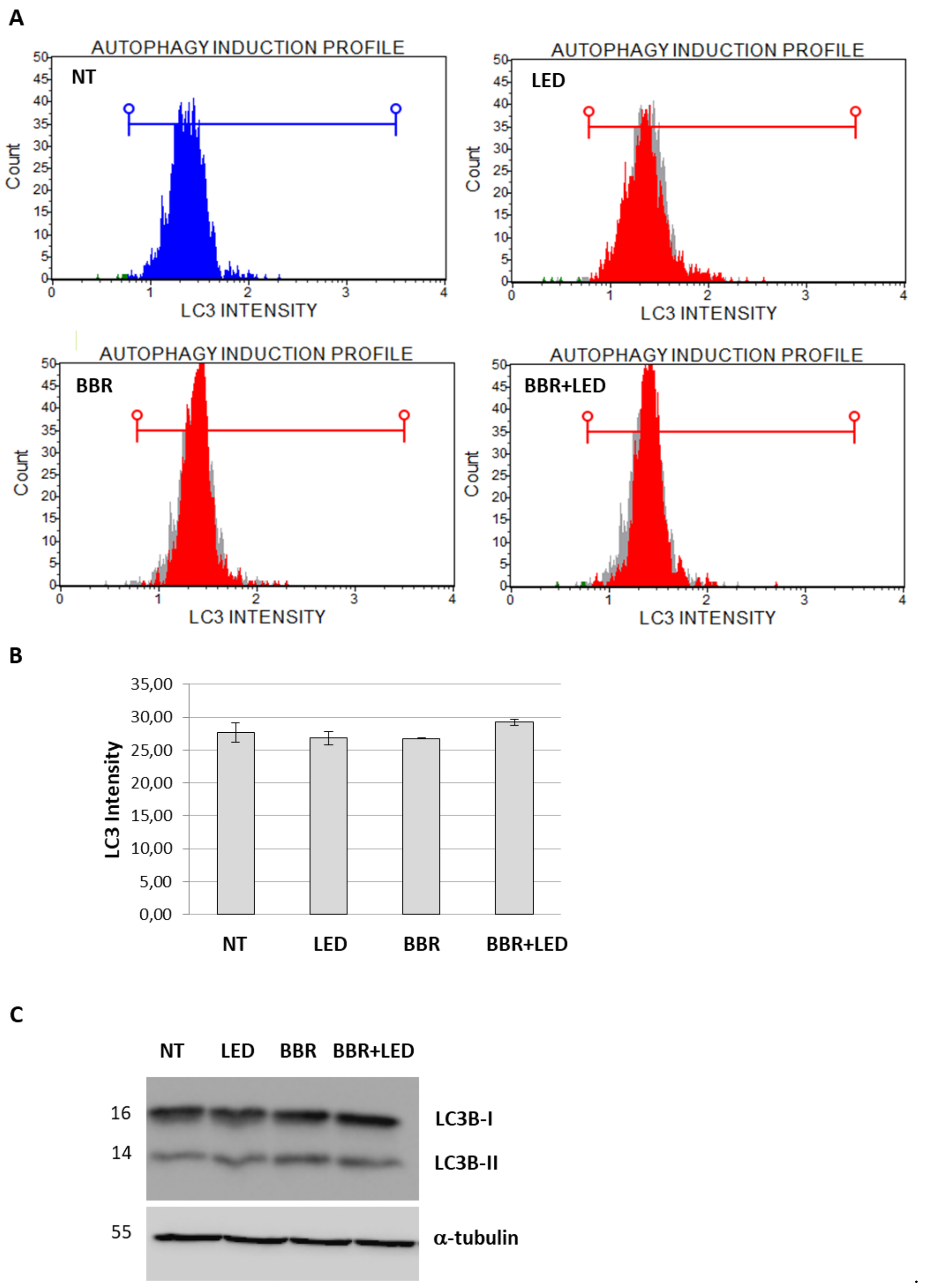

Figure S4. Autophagy evaluation of BBR-PDT treatment. LC3 expression was evaluated in T98G cells as untreated (NT), exposed to led stimulation (LED), BBR (200 µg/ml for 4 hours) administration and with the combined LED+BBR evaluated after 24 hours p.t., following bafilomycin A1 administration (10 nM, at 4 hours before trypsinization). Blue and grey overlapping profiles are related to NT cells (**A**). Trypsinized cells (n=2000) were analysed for LC3 intensity using Muse Autophagy LC3-based kit (Luminex). The histogram of the LC3 intensity of the averages of three independent experiments is reported (**B**) without statistical significance. Immunoblotting analysis of LC3B-II and α-tubulin expression following Bafilomycin A1 administration (10 nM, at 4 hours before trypsinization) (**C**). For each sample, 40 µg of total protein extracts was loaded into 12% polyacrylamide gels. Molecular weights in KDa are reported.

A

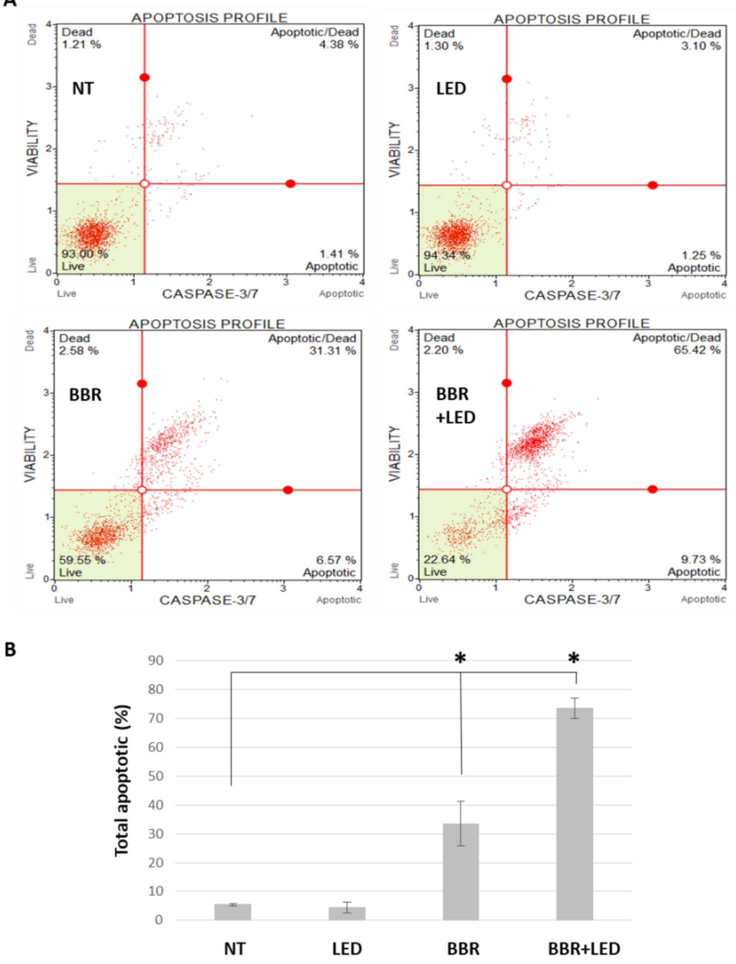

Figure S5. Caspase 3/7 apoptosis profiles. Cytofluorimetric analysis of caspases 3 and 7 activation in T98G cells as untreated (NT), exposed to led stimulation (LED), BBR (200 µg/ml for 4 hours) administration and to the combined BBR+LED treatments) at 24 hours p.t. (**A**). Trypsinized cells were analysed for apoptosis induction using Muse Caspase 3/7 kit (Luminex). The histogram of the Total apoptotic (early+late) cells percentage of the average of three independent experiments is reported (**B**). Asterisks indicated statistical significance compared to untreated cells ($p<0.05$, Anova One-way).

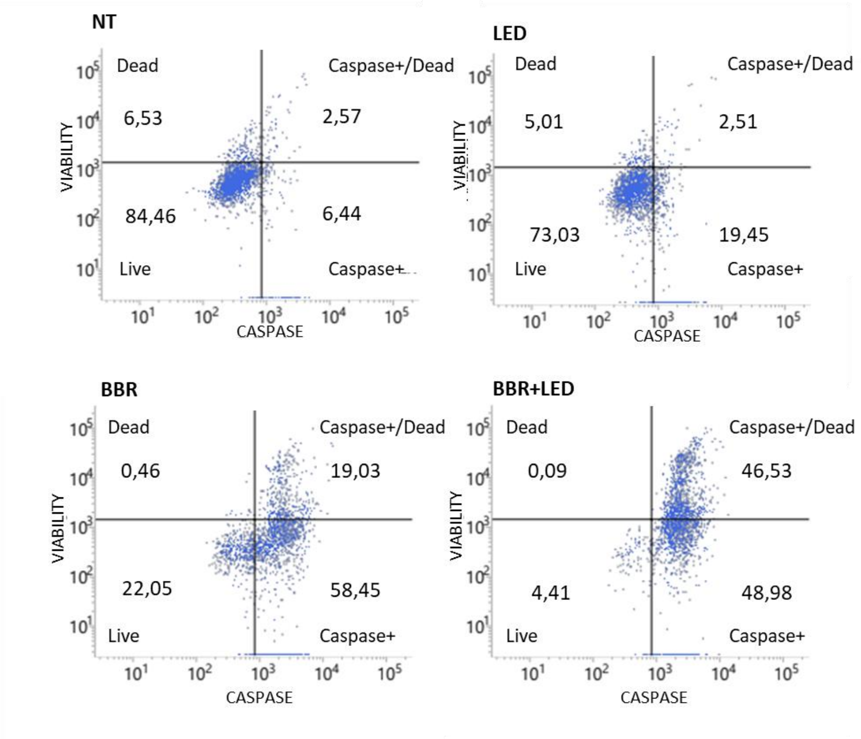

Figure S6. Caspases 1, 3, 4, 5, 6, 7, 8, and 9 apoptosis profiles. Cytofluorimetric analysis of caspases activation in T98G cells as untreated (NT), exposed to led stimulation (LED), BBR (200 µg/ml for 4 hours) administration and with the combined LED+BBR, evaluated after 24 hours p.t.. Trypsinized cells were analysed for apoptosis induction using MultiCaspase kit and subsequently analysed as percentages of cells using a BD Lyric cytofluorimeter (laser source: 488 nm, 586/42 and 700/54 nm bandpass filters).

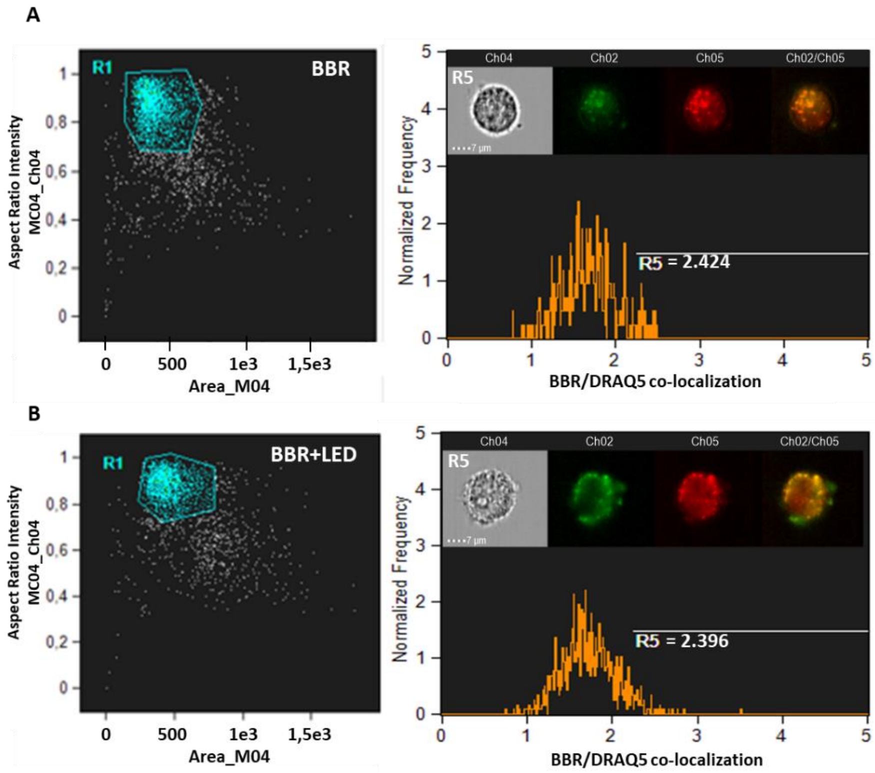

Figure S7. BBR and mitochondria co-localization. T98G cells were treated with BBR (200 µg/ml for 4 hours) (**A**) or with the combined scheme (**B**), both incubated with MitoTracker deep red dye and finally analysed by Amnis ImageStrem flow cytometry evaluating the degree of BBR (Ch02, 488 laser) and MitoTracker (Ch05, 642 laser) co-localization using R5 gate index (Bright Detail R3_MC_Cho2_Cho5 index) according to the «Co-localization» wizard (Ideas). Representative image galleries are reported.
